# Self-administered acupressure for symptom management among Chinese family caregivers with caregiver stress: a randomized, wait-list controlled trial

**DOI:** 10.1186/s12906-016-1409-1

**Published:** 2016-10-28

**Authors:** Agnes Tiwari, Lixing Lao, Amy Xiao-Min Wang, Denise Shuk Ting Cheung, Mike Ka Pui So, Doris Sau Fung Yu, Terry Yat Sang Lum, Helina Yin King Yuk Fung, Jerry Wing Fai Yeung, Zhang-Jin Zhang

**Affiliations:** 1 School of Nursing, Li Ka Shing Faculty of Medicine, The University of Hong Kong, 4/F, William M.W. Mong Block, 21 Sassoon Road, Pokfulam, Hong Kong; 2 School of Chinese Medicine, Li Ka Shing Faculty of Medicine, The University of Hong Kong, 10 Sassoon Road, Pokfulam, Hong Kong; 3 Department of Social Sciences, The University of Hong Kong, 11/F, The Jockey Club Tower, Centennial Campus, The University of Hong Kong, Pokfulam Road, Hong Kong, Hong Kong; 4 Department of Information Systems, Business Statistics and Operations Management, Hong Kong University of Science and Technology, Clear Water Bay, Kowloon, Hong Kong; 5 The Nethersole School of Nursing, The Chinese University of Hong Kong, 6/F, Esther Lee Building, The Chinese University of Hong Kong, Shatin, N.T. Hong Kong; 6 Department of Social Work and Social Administration, The University of Hong Kong, Room 534, Jockey Club Tower, The Centennial Campus, The University of Hong Kong, Pokfulam, Hong Kong; 7 HKSKH Lady MacLehose Centre, No.22, Wo Yi Hop Road, Kwai Chung, New Territories Hong Kong

**Keywords:** Acupressure, Self-administered acupressure, Family caregivers, Caregiver stress, Fatigue, Insomnia, Depression, Health-related quality of life, Intervention, Chinese, Randomized controlled trial (RCT)

## Abstract

**Background:**

Caregiving can be stressful, potentially creating physical and psychological strain. Substantial evidence has shown that family caregivers suffer from significant health problems arising from the demands of caregiving. Although there are programs supporting caregivers, there is little evidence regarding their effectiveness. Acupressure is an ancient Chinese healing method designed to restore the flow of Qi (vital energy) by applying external pressure to acupoints. A randomized, wait-list controlled trial was developed to evaluate the effectiveness of a self-administered acupressure intervention on caregiver stress (primary objective) and stress-related symptoms of fatigue, insomnia, depression, and health-related quality of life (secondary objectives) in Chinese caregivers of older family members.

**Methods:**

Two hundred Chinese participants, aged ≥ 21 years, who are the primary caregivers of an older family member and screen positive for caregiver stress and symptoms of fatigue/insomnia/depression will be recruited from a community setting in Hong Kong. Subjects will be randomized to receive either an immediate treatment condition (self-administered acupressure intervention) or a wait-list control condition. The self-administered acupressure intervention will include (i) an individual learning and practice session twice a week for 2 weeks, (ii) a home follow-up visit once a week for 2 weeks, and (iii) 15-min self-practice twice a day for 6 weeks. The wait-list control group will receive the same acupressure training after the intervention group has completed the intervention. We hypothesize that Chinese family caregivers in the intervention group will have lower levels of caregiver stress, fatigue, insomnia, depression, and higher health-related quality of life after completion of the intervention than participants in the wait-list control group.

**Discussion:**

This study will provide evidence for the effectiveness of self-administered acupressure in reducing stress and improving symptoms of fatigue, insomnia, depression, and health-related quality of life in Chinese family caregivers. The findings will inform the design of interventions to relieve negative health effects of caregiving. Furthermore, the results can raise community awareness and serve as a basis for policymaking, planning, and allocation of resources regarding empowerment of family caregivers for self-care.

**Trial registration:**

Current Controlled Trials NCT02526446. Registered August 10, 2015.

## Background

It is estimated that 1.5 billion people, representing 16 % of the world’s population, will be aged 65 years or older by 2050 [1]. With increasing population age, non-communicable diseases increase in prevalence, including chronic disease and disability. This leads to an increased demand for care, and the primary caregiver role often falls on family members [2]. In this study, the primary caregiver of an older family member is defined as one who provides unpaid care to the care recipient for no less than 14 h per week, and the care recipients are older family members (aged ≥ 65 years) irrespective of their health problems/disabilities.

While caring for an older family member can be rewarding for some, evidence shows that family caregivers are at risk of emotional, mental, and physical health problems arising from the complexity and strains of caregiving [2–5]. Caregiver stress, defined as “the burden or strain that caregivers face when caring for a person with a chronic disease,” is prevalent and associated with stress-related symptoms, notably fatigue, insomnia, and depression [3]. Fatigue is often the initial and most difficult problem resulting from the stressful caregiving process, and leads to sleep disturbance, anxiety, and depression [6]. It is estimated that more than one-third of family caregivers suffer from poor health [7].

Not only does caregiver stress put family caregivers at risk for poor health outcomes, it may also affect their quality of life [8] and hinder their ability to provide care, with negative consequences for their care recipients [9]. The adverse impact of caregiver stress on the health and safety of both caregivers and care recipients can lead to increased social and healthcare costs [2]. Despite the recognition that stress-related health symptoms among family caregivers is a public health priority [1], there is little evidence about the effectiveness of symptom management for these caregivers.

We aim to study Chinese family caregivers because China comprises 19 % of the world’s population and is expected to have an older population of 25 % by 2030 [10]. Furthermore, with 90 % of older Chinese living at home [11], much of the caregiving responsibilities are likely to fall on their family members. Although the Confucian value of filial piety may buffer the demands of caregiving, Chinese family caregivers are not exempt from caregiver stress [12]. Therefore, these caregivers, similar to their Western counterparts, are in need of effective, achievable, and acceptable interventions to help them manage caregiver stress.

### Acupressure and symptom management

Acupressure, defined as the application of pressure on acupoints using the hands, fingers, or thumbs [13–15], is a non-invasive technique based on the meridian theory of Traditional Chinese medicine (TCM). TCM theory holds that meridians, which are channels in a network of energy pathways throughout the body, regulate the flow of Qi (vital energy) and the unbalanced flow of Qi results in disease [16]. By applying pressure to acupoints (trigger or active points) on the surface of the skin, acupressure stimulates the meridians, resulting in the opening of the channels and balancing of energy, thus restoring health [14, 16]. Because acupressure uses the application of pressure to acupoints without penetrating the skin, it is non-invasive and painless [17, 18]. The use of acupressure for positive symptom management in healthy people and patients by trained practitioners has been reported [17–22].

In addition to the administration of acupressure by trained practitioners, self-administered acupressure has also been used for symptom management. Self-administered acupressure is acupressure performed by the recipients themselves after undergoing appropriate training. This technique has a number of advantages including flexibility, low cost, and empowerment [18]. Studies have reported on the clinical application of self-administered acupressure [14, 23]. In addition, systematic reviews of the effect of self-administered acupressure for symptom management have been conducted. These reviews included perceived stress, insomnia, and sleep disturbances, and the studies found positive effects and safety. However, attention was also drawn to the need for well-designed randomized controlled trials [13, 18].

Self-administered acupressure is likely to suit the family caregivers because their caregiver responsibilities often leave them with little time and flexibility to seek their own treatment. Once they have mastered the technique of self-administered acupressure, they can choose when and where to conduct the intervention to suit their caregiver activities and their own needs.

In the present study protocol, we detail a self-administered acupressure intervention protocol for Chinese family caregivers with caregiver stress based on our previous study on: (i) a similar model of self-administered acupressure previously tested in women with osteoarthritic knee pain that was shown to be feasible and safe [14]; (ii) factors aggravating or buffering caregiver stress among Chinese family caregivers [12]; (iii) a case management approach to improve the health outcomes of Chinese family caregivers of dementia patients [24]; and (iv) the scientific basis of symptom management [25]. In light of the needs of these caregivers and the little evidence on the effectiveness of self-administered acupressure in symptom management, a specifically designed self-administered acupressure intervention for Chinese family caregivers will be implemented and evaluated in this study.

### Aims and hypotheses

The primary aim of this randomized, wait-list controlled trial is to evaluate the effectiveness of a self-administered acupressure intervention on caregiver stress among Chinese caregivers of older family members. The secondary aim is to evaluate the effectiveness of the self-administered acupressure intervention on the Chinese family caregivers’ stress-related symptoms of fatigue, insomnia, and depression and their health-related quality of life.

We hypothesize that, on completion of a self-administered acupressure intervention, and as compared with the wait-list control group, Chinese family caregivers in the intervention group will have:

(i) lower levels of caregiver stress, as measured by the Caregiver Burden Inventory;
(ii) lower scores of fatigue symptoms, as measured by the Piper Fatigue Scale;
(iii) lower scores of insomnia symptoms, as measured by the Pittsburgh Sleep Quality Index;
(iv) lower scores of depression symptoms, as measured by the Patient Health Questionnaire; and
(v) higher scores of health-related quality of life, as measured by the SF-12 Health Survey.

## Methods/Design

This is a randomized, wait-list controlled trial. There will be two groups: an intervention group and a wait-list control group. The participants randomly assigned to the intervention group will receive an immediate treatment condition (the self-administered acupressure intervention), while those assigned to the wait-list control group will receive a wait-list control condition (the same self-administered acupressure intervention but after the intervention group has completed the treatment condition). The design allows all participants to receive the intervention eventually but at the same time also controls the confounding variables that could cause spurious causality.

### Participants

A total of 200 participants will be recruited for the study. Chinese family caregivers will be eligible to participate if they meet all of the following criteria:

1. Chinese men or women, 21 years of age or older, able to communicate in Cantonese or Putonghua. Justifications: an ability to command the Chinese language (Cantonese or Putonghua) is essential because the acupressure protocol is written and conducted in Chinese. Furthermore, the intervention requires participants to have the self-discipline for compliance, hence the decision to select more mature participants of age ≥ 21 years.
2. Primary caregiver of an older family member aged ≥ 65 years. Justifications: the literature has shown that caring for an older family member is a key source of caregiver stress, hence our decision to adopt a more inclusive approach to target older family members who are the care recipients, irrespective of their health problems/disabilities. We recognize that the older care recipients likely have different health problems/disabilities, which may affect their dependency on the caregivers and the level of caregiver stress. Therefore, we will take into account the care recipients’ health problems/disabilities in the data analysis.
3. Providing unpaid care to the care recipient at no less than 14 h per week. Justification: this criterion will exclude paid or occasional caretakers whose needs and caregiver stress, if any, are likely to be different from that studied in this project.
4. Primarily responsible for making day-to-day decisions and providing assistance to the care recipient in tasks relating to activities of daily living (e.g., bathing, dressing, and toileting) and/or instrumental activities of daily living (e.g., housework, grocery shopping, preparing meals, and managing medications). Justification: this criterion will exclude those who are not the primary caregivers.
5. Screened positive for caregiver stress (a summed score of ≥ 25 as measured by the Caregiver Burden Inventory), with symptoms of fatigue (a mean score of ≥ 4 as measured by the Piper Fatigue Scale), insomnia (a global score of > 5 as measured by the Pittsburgh Sleep Quality Index), or depression (a total score of ≥ 10 as measured by the Patient Health Questionnaire). Justifications: caregivers with caregiver stress are the participants targeted while fatigue, insomnia, and depression are the outcome measures in the proposed study.

Chinese family caregivers will be excluded if they have:

1. Cognitive impairment (a Mini Mental State Examination score of ≤ 23). Justification: cognitive impairment will interfere with their comprehension of the intervention.
2. Major chronic illness (e.g., cancer) or are currently taking medication (e.g., opiates) that may prevent them from performing the intervention. Justification: they may have difficulty completing the intervention.
3. Participated in interventional studies involving acupressure or acupuncture previously. Justification: their prior experience may affect their response to the proposed intervention.

### Sample size

The power calculation is based on the test for significant difference between the mean pre-post differences of the caregiver stress scores in the intervention and control groups. Because no study has used self-administered acupressure as an intervention to reduce caregiver stress, we determined our study sample size based on the findings of a previous study measuring caregiver burden of those caring for community-residing patients with Alzheimer’s disease [24]. This study reported the mean and standard deviation of the CBI score to be 47.54 and 17.61, respectively. Assuming a moderate correlation ($p$ = 0.7) between the pre and post intervention CBI scores, we approximate the pooled standard deviation of a two-sample *t*-test as $17.61\sqrt{2\left(1-p\right)}=13.64$. Taking a clinical difference of *d* = 6, which is considered a 10–15 % improvement owing to intervention (using the mean 47.54 as a reference) and a Type I error rate of 5 %, *n* = 83 was determined to have a power greater than 80 %. Assuming an attrition rate of 15 %, the target sample size is at least 98 per group. We rounded up the number of participants to 100 in each group, which makes a total of 200 participants.

### Setting

The proposed trial will be conducted in a nongovernment organization (NGO) in Hong Kong. The NGO has more than 40 outreach centers covering three densely populated districts with an older population of around 14 %. This is comparable to the average older population of 13.5 % in Hong Kong [26]. The NGO has been providing caregiver support and older services in the districts for several decades.

### Recruitment

A flyer with a brief description of the present project and an invitation to participate will be displayed in the host NGO center and its outreach sites. An advertisement will also be placed in the NGO’s newsletters. Additionally, promotional sessions will be conducted during the activities for family caregivers organized by the NGO. Potential participants who express an interest will be referred by the center staff to our research team. The research assistant responsible for recruitment will contact the individual and provide information about the project together with the rights as a research subject and the voluntary nature of the participation. If the person agrees to participate, a written consent form will be signed. After obtaining informed consent, an assessment of eligibility will be made according to the inclusion and exclusion criteria as described above. Those assessed to be not eligible will be thanked for their interest and no further contact will be made. Those who meet the inclusion criteria will be enrolled in the study.

### Randomization and blinding

Eligible participants will be randomly assigned to either the intervention group (*n* = 100) or the wait-list control group (*n* = 100) using a computerized blocked randomization scheme operated by the study programmer (SP) who is not involved in the recruitment. A series of random numbers will be generated to determine the group assignment of the participants. The group assignment results will be kept in separate, sealed, opaque envelopes. The entire randomization process will be securely conducted by the SP and the group assignment of participants will be centrally controlled. Neither the research assistant conducting the recruitment, nor the participant will know the group assignment until the envelope is opened.

Questionnaires will be completed by each of the participants and numerically coded to ensure that the group allocation of the participant is not revealed. The numerical codes and the names of the participants will be stored separately and securely in the central office. Researchers conducting data collection and analysis will be blinded to the group allocation of the participants.

### Intervention

The intervention, 28 h in total, extends over an 8-weeks period and includes: (i) individual learning and practice (1^st^–2^nd^ wk): a one-time 1-h introduction and ice-breaking exercise at the start of the first session to be followed by a 1-h training session on self-administered acupressure provided by certified trainers in the participant’s home twice a week for 2 consecutive weeks (total, 5 h); (ii) home follow-up (3^rd^–4^th^ wk): a 1-h home visit by certified trainers to reinforce learning and self-practice once a week for 2 consecutive weeks (total, 2 h); and (iii) self-practice (3^rd^–8^th^ wk): self-administered acupressure by the participant at home, to be undertaken not less than one h after a meal, for 15 min twice a day for 6 weeks following the completion of the 2-weeks training session (total, 21 h).

For each of the 1-h training sessions during the first 2 weeks, a brief introduction to the basic theories of TCM and acupressure therapy will be provided (15 min). The introduction will be followed by a demonstration of self-administered acupressure by the trainers (15 min). Self-administered acupressure will then be practiced, until proficient, by the participant under the guidance of the trainers (30 min). The trainers, made up of a senior year TCM student and a senior year nursing student, will be trained and certified by a licensed TCM practitioner from the researchers’ School of Chinese Medicine.

For monitoring of compliance, during the 5^th^ through 8^th^ weeks of the intervention, once a week, phone calls will be made by the same team of trainers to remind the participant to perform the self-administered acupressure with feedback for any questions or expressed concerns. Any medical incidents (e.g., visits to hospital or doctor for caregiver health problems) will be documented. Each participant will use an “acupressure” diary to record the frequency, duration, and time of the acupressure conducted each day, and the record will be checked by the trainer during the telephone calls.

Training materials will be provided to each participant. The self-administered acupressure protocol (Table 1), a poster illustrating the acupoints, and stickers used to label each acupoint will be provided to the participant at the first training session. Acupoint selection will be demonstrated step-by-step by the trainers, and the participants will be asked to find the selected acupoints by themselves under the guidance of the trainers. Once they have mastered the location of the selected acupoints, the participants will be guided, with assistance from the trainers as required, to perform the self-administered acupressure as described in the protocol. An audio-recorded step-by step procedure of the self-administered acupressure will be provided to the participants for reinforcement of learning. The audio recording, protocol, posters, and stickers are designed to enhance accuracy and compliance during home practice.

**Table 1:** Standard protocol- self-administered acupressure for symptom management

Sequence/acupoint	Location		Function	How-to-do	Frequency (Times/duration)
1.Baihui (GV20, 百會)	On the vertex of the head at the sigittal midline of the scalp at the midpoint of the line connecting the apexes of both ears		Treatment of various mental disorders, in particular insomnia, depression, anxiety, headache and decreased memory	Using 4 finger pads gently tap the area of this acupoint on the scalp	60/1 min
2.Fenchi (GB20, 風池)	On the nape, in a depression between the upper portion of the sternocleidomastoid muscle and the trapezius		A commonly used point for acupressure to treat headache, neck and shoulder pain and stiffness. Also beneficial in relieving convulsion, agitation, insomnia and stress-related symptoms	Using two thumbs press on the points bilaterally while the other four fingers should hold the back of the head naturally	60/1 min
6.Hegu (LI4, 合谷)	On the dorsum of the hand, between the 1st and 2nd metacarpal bones, in the middle of the 2nd metacarpal bone on the radial side		Expels Wind and releases the exterior, tonifies qi and strengthens immunity; used to manage every type of pain and psychogenic tense	Using thumb pad firmly massage the surrounding area of this acupoint on the dorsum of the hand unilaterally	30/1 min for each side
4.Shenshu (UB 23, 腎俞)	On the low back at 1.5 cun lateral to the posterior midline at the level of the 2nd lumbar vertebral spine		Well known For all kidney related issues which affect the brain, bone, hair, teeth and/or hearing. Useful for deficiency conditions: exhaustion, weakness, chronic fatique, good point for the elderly as Kidney Jing is naturally depleted	Using the fists gently tap the lumbar area of this acupoint at the low back bilaterally	60/1.5mins
5.Zhongwan (CV12, 中脘)	On the **upper abdomen** and on the anterior midline, 4 cun above the centre of the umbilicus.		Innervated by the spinal nerves originating from the same segments of the spinal cord that sends visceral nerve fiber innervating the stomach. Can improve digestive function and relieve abdominal distention/pain, constipation and insomnia	Using finger pads in clockwise circle gently massage the upper abdomen area, 4 cun above the umbilicus	200/2mins
6.Qihai (CV6, 氣海) GuanyYuan (CV4, 關元)	On the **lower abdomen** on the anterior midline at 1.5 cun and 3 cun below the centre of the umbilicus, respectively.		Often applied together in acupressure to tonify Qi because Essential Qi (元氣) is housed and circulated in these two points. Beneficial for constipation, retention of urination, frequent nocturia and indigestion. Modulate the limbic-medial prefrontal network related to cognitive function	Using finger pads in clockwise circle gently massage the lower abdomen area, 3 cun below the umbilicus	200/2mins
7.Zusanli (ST36, 足三里)	On the anterior lateral side of the leg at 3 cun below the knee joint, one middle finger breadth from the anterior crest of the tibia.		Broad therapeutic effects, from gastro, intestinal and endocrinal diseases to neuropsychiatric disorders. Stimulation at Zu-San-Li evokes the robust response in limbic-paralimbic-neocortical network involved in autonomic, pain, mood and cognitive function	Using thumb pad firmly massage the area bilaterally on the anterior lateral side of the leg, 3 cun below the knee joint	60/1.5mins for each side
8.Yongquan (KD1,涌泉)	On sole, in a depression with foot in plantar flexion, at the junction of the anterior 1/3 and posterior 2/3 of line connecting base of the 2nd and 3rd toes with the heel		Useful for headaches, hypertension, low back pain, insomnia, palpitations, anxiety, poor memory, mania, hot flashes, night sweats, and loss of consciousness or yang collapse	Using 4 finger pads firmly massage the area bilaterally on each sole, in depression with foot in plantar flexion	100/2.5mins for each side

Consent to publish the images in the table has been obtained from the patients featured

### Instruments

The following study instruments (with the exception of the Demographic Questionnaire) will be administered at four time points: (a) pre-intervention (T0, baseline), i.e., on entry to study after randomization but before intervention; (b) post-training (T1, end of 2^nd^ week), i.e., on completion of the 2-weeks individual learning and practice; (c) post-intervention (T2, end of 8^th^ wk), i.e., on completion of the 8-weeks self-administered acupressure intervention; and (d) follow-up (T3, end of 12^th^ wk), i.e., 4 weeks after the completion of the intervention.

- The Chinese version of the Caregiver Burden Inventory (C-CBI, 24 items) [27] will be used to (i) initially screen potential participants for caregiver stress, and (ii) assess levels of caregiver stress at different time points in the study. The C-CBI has been validated for the Chinese population and demonstrated satisfactory internal consistency (Cronbach’s alpha 0.9) [27]. Each item is assessed using a 5-point Likert scale ranging from 0 (never) to 4 (nearly always). For the initial screening, participants with a summed score of ≥ 25 will be identified as experiencing caregiver stress and recruited into the study.
- The Chinese version of the Piper Fatigue Scale (C-PFS, 22 items) will be used to (i) initially screen potential participants for the symptom of fatigue, and (ii) assess the levels of fatigue experienced by the participant at different time points in the study. The C-PFS has been validated with good reliability (Cronbach’s alpha 0.93) [28]. Each item is assessed using a numeric scale of “0” to 10” with higher scores representing more fatigue. For the initial screening, participants with a mean score of ≥ 4 will be identified as experiencing fatigue and recruited into the study.
- The Chinese version of the Pittsburgh Sleep Quality Index (C-PSQI, 19 items) will be used to (i) initially screen potential participants for the symptom of insomnia, and (ii) assess the levels of insomnia experienced by the participant at different time points of the study. The C-PSQI has been validated with good reliability (Cronbach’s alpha 0.86) [29]. Insomnia (sleep disturbances in subjective sleep quality, sleep latency, sleep duration, habitual sleep efficiency, sleep disturbances, use of sleeping medication, and daytime dysfunction) will be assessed on a 4-point Likert scale ranging from 0 (no difficulty) to 3 (severe difficulty). For the initial screening, participants with a global (summed) score of > 5 will be classified as experiencing insomnia and recruited into the study.
- The Chinese version of the Patient Health Questionnaire (C-PHQ, nine items) will be used to (i) initially screen potential participants for the symptom of depression, and (ii) assess the levels of depression experienced by the participant at different time points of the study. The C-PHQ is one of the most popular self-administered screening tools for the symptom of depression that has been validated in the Hong Kong Chinese population [30]. Each item is scored from 0 (not at all) to 3 (nearly every day), with a total score ranging from 0 to 27. Cutoff values of 5, 10, 15, and 20 have been widely used to define mild, moderate, moderately severe, and severe depressive symptoms. For the initial screening, participants with a total score of ≥ 10 will be identified as experiencing the symptom of depression and recruited into the study.
- The Chinese version of the SF-12 version 2 Health Survey (C-SF-12v2, 12 items) has demonstrated validity and equivalence for Chinese populations [31] and will be used to assess health-related quality of life. The 12 items are grouped under the mental component summary and physical component summary. The survey is scored by recoding the items, computing the raw scale scores, and transforming the scores to a range from 0 to 100 according to the standard scoring algorithm. Higher scores indicate a better health status.
- Health economics assessment (HEA) will be assessed to determine the cost minimization [32] related to the intervention, with items on: (i) number of physician visits, (ii) use of prescription drugs, and (iii) incidence of inpatient hospitalization.
- A demographic questionnaire (DQ) will be used to collect information on age, education level, marital status, number and age of children, employment status, financial hardship, number of care recipients, total number of hours of caregiving per week (for the older adult care recipient and children, if appropriate), length of caring (years/months of caregiving), assistance received from other family members (including number of people providing care), paid/occasional caregivers, receipt of comprehensive social security assistance, need for financial support, and number of years living in Hong Kong. Information will also be collected regarding the care recipient’s health and dependency including age, health problems, disabilities, and degree of dependency on the caregiver.

### Procedures

**Fig. 1:**
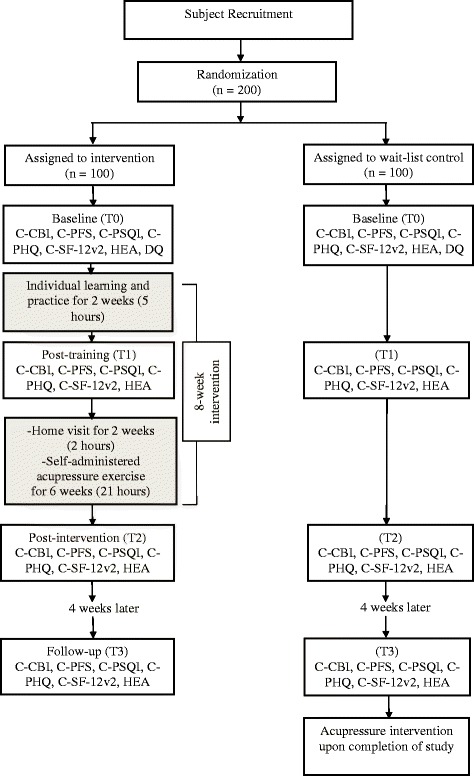
Flow diagram of intervention/wait-list control and data collection points

Upon study entry, participants in both groups will be asked to complete the Chinese version of the questionnaires (T0, baseline assessment), including C-CBI for caregiver stress, C-PFS for fatigue, C-PSQI for insomnia, C-PHQ for depression, C-SF-12v2 for quality of life, HEA for cost minimization, and DQ for participant profile. The participants assigned to the intervention group will then receive the intervention as described. When the intervention group reaches post-training, post-intervention, and follow-up time points (T1, T2, and T3, respectively), participants in both groups will complete C-CBI, C-PFS, C-PSQI, C-PHQ, C-SF-12v2, and HEA. After the completion of data collection at T3, participants in the wait-list control group will then receive the self-administered acupressure training. The immediate treatment received by the intervention group, the wait-list control condition received by the control group, and the data collection points are shown in Fig. 1.

### Data analysis

The primary outcome is caregiver stress and the secondary outcomes are symptoms of fatigue, insomnia, depression, and health-related quality of life.

The effectiveness of the self-administered acupressure intervention on caregiver stress, symptoms of fatigue, insomnia and depression, and health-related quality of life will be assessed. To do this, the scores of the C-CBI, C-PFS, C-PSQI, C-PHQ-9, SF-12v2, and HEA collected at four different time points will be analyzed for changes from baseline (T0) to the post-intervention (T2) and from the post-intervention (T2) to the follow-up (T3).

For the primary analysis, the levels of caregiver stress on completion of the intervention (T2) between the intervention and wait-list control groups will be assessed by a regression analysis with adjustment of baseline values and accounting for any possible effect of the demographics. Residuals will be checked to ensure adequacy of the method. In addition, changes in caregiver stress from baseline (T0) to follow-up (T3) will be assessed by paired *t*-tests. The intention-to-treat principle will be adopted and all study subjects will be included in the analysis with missing values replaced by the last observed values or imputed by regression substitution.

For secondary analysis, the scores of the C-PFS, C-PSQI, C-PHQ, C-SF-12v2, and HEA will be compared for differences between the intervention and wait-list control groups by a linear mixed effects model with the baseline value of the scale and the intervention group as fixed factors and the intercept as a random factor. Moreover, effects of the demographics, care recipients’ health problems/disability, and dependency on the outcomes will be explored by considering them as fixed factors in the linear mixed effects model. Changes in mean scores from baseline will also be assessed by a linear mixed effects model with the use of linear contrasts. Multivariate analysis will also be conducted to simultaneously study changes in clusters of mean scores.

Baseline characteristics (T0) between the intervention and wait-list control groups will be assessed by chi-square test and Mann–Whitney U test for categorical and continuous data, respectively.

Statistical significance is defined as *p* < 0.05 with a two-sided test. All statistical analyses will be conducted with Statistical Package for the Social Sciences (SPSS) program.

## Ethics, consent and permissions

This study protocol was approved by the Institutional Review Board of the University of Hong Kong/Hospital Authority Hong Kong West Cluster (HKU/HA HKW IRB: UW 15–367) on June 26, 2015. The study will be conducted according to the Declaration of Helsinki. Participation in the study is entirely voluntary. An information sheet is provided and a written consent is required from all participants. If participants choose to withdraw from this study, they may do so at any time with no questions asked.

## Discussion

This study protocol describes the implementation and evaluation of a self-administered acupressure intervention for Chinese family caregivers with caregiver stress. This will be the first randomized controlled trial to test the effectiveness of self-administered acupressure in reducing the stress of caregivers and improving their stress-related symptoms of fatigue, insomnia, depression, and health-related quality of life.

The emotional and physical strain of caregiving and its adverse impact on family caregivers’ health and well-being is recognized as a serious public health problem [3, 33, 34]. Although a variety of pharmacological and psychosocial interventions have been developed to alleviate caregiver stress, the therapeutic benefits are modest [35–37]. Therefore, there is a need to find interventions that are not only effective but also acceptable to caregivers. Providing interventions to these caregivers is challenging; not only may their motivation be hampered by the stress of never-ending caregiving responsibilities, but their needs often have to take second place after those of their care recipients. If proven effective, the self-administered acupressure intervention would open up an attainable avenue for family caregivers to release their caregiving stress in a safe, feasible, and affordable manner. By reducing the burden of caregiver stress, it is intended that the self-administered acupressure intervention will not only alleviate health declines, but also lower health and social care costs for these caregivers and their care recipients. Furthermore, the realization of what they can achieve in promoting health and well-being through self-administered intervention may also empower the caregivers to make optimal lifestyle choices.

This study has the potential of informing health and social care providers about the design and implementation of interventions to buffer the adverse effects of caregiver stress. In addition, the trial findings will also provide the much-needed evidence to apprise policy-makers of the need for socioeconomic policies to more effectively empower family caregivers to take care of themselves and their care recipients.

## Abbreviations

C-CBI: Chinese version of the Caregiver Burden Inventory
C-PFS: Chinese version of the Piper Fatigue Scale
C-PHQ: Chinese version of the Patient Health Questionnaire
C-PSQI: Chinese version of the Pittsburgh Sleep Quality Index
C-SF-12v2: Chinese version of the SF-12 version 2 Health Survey
DQ: Demographic questionnaire
HEA: Health economics assessment
NGO: Nongovernment organization
RCT: Randomized controlled trial
SP: Study programmer
SPSS: Statistical Package for the Social Sciences
TCM: Traditional Chinese medicine
